# Association of baseline level of cardiovascular risk burden and its temporal changes with cognitive decline

**DOI:** 10.3389/fnagi.2022.895188

**Published:** 2022-09-01

**Authors:** Xiaoli Ji, Hui Gao, Daoyuan Sun, Wensui Zhao, Jianlin Zhuang, Kan Wang, Fariba Ahmadizar

**Affiliations:** ^1^Department of Occupational Disease, Shanghai Pulmonary Hospital, Tongji University School of Medicine, Shanghai, China; ^2^Changning Center for Disease Control and Prevention, Shanghai, China; ^3^Department of Epidemiology, Erasmus Medical Center, Rotterdam, Netherlands; ^4^Department of Data Science and Biostatistics, University Medical Center Utrecht, Utrecht, Netherlands

**Keywords:** Framingham General Cardiovascular Risk Score, change, cognitive decline, older people, cohort

## Abstract

**Background and aim:**

Previous studies on cardiovascular risk burden assessed by the Framingham General Cardiovascular Risk Score (FGCRS) and cognitive trajectories mainly focus on Western populations and most of them have used a single measure of cardiovascular risk. In this study, among middle-aged and older Chinese, we investigated (i) the association of baseline FGCRS with subsequent cognitive decline and (ii) the association of FGCRS change with concomitant cognitive decline.

**Materials and methods:**

In wave 1 to wave 4 (2011–2018) of the China Health and Retirement Longitudinal Study, global cognition was assessed by orientation, memory, and executive function. FGCRS was assessed and categorized into tertiles (low, intermediate, and high) at baseline (2011) and 4 years after (2015). Furthermore, external validation was performed to check its generalizability using the English Longitudinal Study of Ageing (ELSA) 2008–2018.

**Results:**

In total, 6,402 participants with a mean [standard deviation (SD) age of 57.8 (8.4) years, 49.0% women] with complete baseline data and at least one reassessment of cognitive function were included. A 10% increment in baseline FGCRS was associated with a faster decline in global cognition (−0.010 SD/year, 95% CI −0.013, −0.008). Among 4,336 participants [mean (SD) age of 57.8 (8.2) years, 50.0% women] with data on FGCRS changes, compared to individuals with the consistently low FGCRS (reference group), a faster global cognition decline rate was observed in the low to intermediate group (−0.026 SD/year, 95% CI −0.045, −0.007), the low to high group (−0.052 SD/year, 95% CI −0.102, −0.001), the consistently intermediate group (−0.019 SD/year, 95% CI −0.033, −0.005), the intermediate to high group (−0.040 SD/year, 95% CI −0.058, −0.022), the high to intermediate group (−0.024 SD/year, 95% CI −0.047, −0.002), and the consistently high group (−0.047 SD/year, 95% CI −0.060, −0.034). Similar trends were observed for individual cognitive domains. Results from the external validation using the ELSA remained consistent.

**Conclusion:**

Higher baseline FGCRS was associated with faster cognitive decline. However, there was no consistent relationship between the direction of changes in FGCRS and cognitive decline.

## Introduction

Dementia is the leading cause of disability and dependency among the elderly. According to the Gauthier et al. (2021), over 55 million people live with dementia, which is projected to reach 78 million by 2030 (Gauthier et al., 2021). Due to the lack of effective treatment, identifying modifiable risk factors for cognitive decline, a prodromal feature of dementia, has become an important strategy to halt the dementia epidemic (Gorelick et al., 2017; World Health Organization, 2019; Livingston et al., 2020). The detrimental effect of traditional cardiovascular risk factors, including smoking, hypertension, and diabetes on cognitive function, has been well established (Baumgart et al., 2015). Still, these risk factors are interrelated, making it difficult to isolate their individual effects (Qiu and Fratiglioni, 2015; Armstrong et al., 2019). Also, given the multifactorial aetiology of cognitive decline and dementia, multidomain interventions that target several risk factors simultaneously might be necessary for an optimal preventive effect (Kivipelto et al., 2018).

The Framingham General Cardiovascular Risk Score (FGCRS), calculated using the information on age, total cholesterol, high-density lipoprotein (HDL) cholesterol, systolic blood pressure, use of blood pressure-lowering medication, smoking, and diabetes, was originally developed to assess general cardiovascular risk burden (D’Agostino et al., 2008). To date, many studies have investigated the association between FGCRS and cognitive decline, but mainly focusing on populations in Western countries (Harrison et al., 2014). Given the ethnic differences in the effects of these risk factors on cognitive function (Lipnicki et al., 2017; Volgman et al., 2018), this evidence may not be generalizable to the Chinese population. In addition, despite their prospective nature, former studies used a single assessment of FGCRS; whether and how the FGCRS changes over time are related to cognitive decline is largely unknown.

The China Health and Retirement Longitudinal Study (CHARLS) is a nationally representative aging cohort with large sample sizes and repeated cognitive assessments. Here we used it to investigate (i) the association of baseline FGCRS (measured at wave 1) with subsequent cognitive decline (wave 1 to wave 4); and (ii) the association of FGCRS change (measured between wave 1 and wave 3) with concomitant cognitive decline (wave 1 to wave 4).

## Materials and methods

### Study design and population

**FIGURE 1:**
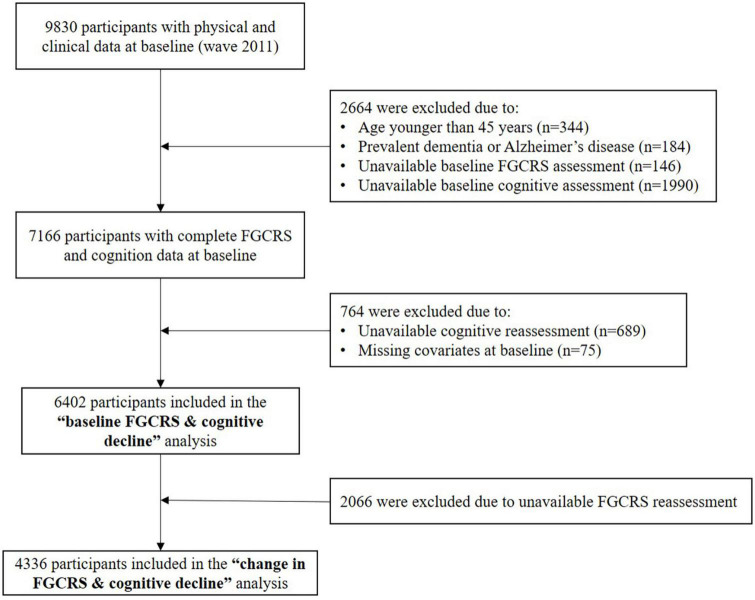
Flow chart of the study participants.

In this study, we used data from wave 1 to wave 4 (2011 to 2018) of the CHARLS, a community-based longitudinal cohort conducted in China. The detailed study design has been described elsewhere (Zhao et al., 2014). The flow chart for participants’ selection of the present study is shown in Figure 1. Briefly, a total of 9,830 participants had physical and clinical data at baseline; 3,428 were excluded for the following reasons: younger than 45 years (*n* = 344), self-reported doctor-diagnosed mental disease (e.g., dementia, Alzheimer’s disease, or cognitive problem) (*n* = 184), unavailable information to assess FGCRS (*n* = 146), or cognitive function (*n* = 1990) at baseline, loss to follow-up (*n* = 689), or missing covariates at baseline (*n* = 75). Hence, 6,402 participants were included with complete measurements of FGCRS and cognitive function at baseline and at least one reassessment of cognitive function during 7 years of follow-up. Of these, 4336 participants also had data about FGCRS at wave 3 (4 years after baseline).

### Measurements

Structured questionnaires were administered by trained field workers using a computer-assisted personal interview system to collect demographic, lifestyle, and medical history data. Body mass index (BMI) was calculated as body weight divided by the square of height (kg/m^2^). A high level of education was defined as completing at least the senior level of high school. Smoking status was recorded and dichotomized into never/former *vs.* current. The 10-item CES-D Scale was used and participants scoring higher than 12 were defined as having depressive symptoms. Blood pressure level was measured three times at a sitting resting position. The average of the second and third blood pressure readings, or the average of the first and second blood pressure readings if the third reading was unavailable, was used for the analyses. Information about blood pressure- or blood glucose-lowering medications and self-reported doctor-diagnosed cardiovascular disease (heart disease and stroke) were also registered.

Blood samples were collected every two waves with details of the process described elsewhere (Chen et al., 2019). Participants were asked to fast overnight before collection. Blood glucose, total cholesterol and HDL cholesterol were assessed with routine clinical chemistry methods. Glycated hemoglobin (HbA1c) was measured using the high-performance liquid chromatography method. For the current study, we used the blood biomarker data measured at baseline (wave 1) and 4 years after (wave 3).

### Framingham General Cardiovascular Risk Score

Type 2 diabetes was defined based on the 2020 American Diabetes Association criteria (American Diabetes, 2020) as fasting blood glucose ≥ 7.0 mmol/L (126 mg/dL), non-fasting blood glucose ≥ 11.1 mmol/L (200 mg/dL), HbA1c level ≥ 47.5 mmol/mol (6.5%), self-reported doctor-diagnosed diabetes, or current use of blood glucose-lowering medications. We calculated FGCRS based on age, total cholesterol, HDL cholesterol, systolic blood pressure, blood pressure-lowering medication, smoking, and diabetes for each participant at baseline and 4-years after. A higher FGCRS indicates a greater risk of future cardiovascular events.

### Cognitive assessments

Cognitive assessment was performed in all waves, including three domains: orientation, memory, and executive function, with higher scores indicating better cognitive function (Ma et al., 2020). Orientation was measured by asking four questions regarding the date (year, month, day of month, and day of week). One point was given for each correct answer, with the sum score ranging from 0 to 4. The memory assessment comprised immediate and delayed recall for ten unrelated words. The sum of words successfully recalled in these two tests was used and ranged from 0 to 20. For the assessment of executive function, the participant was asked to observe and draw a picture of two overlapping pentagons (three points were given for a successful drawing and 0 points for an unsuccessful drawing) and also to do the serial seven test, in which the participant was asked to serial subtraction of 7 from 100 (up to five times). The executive function score was the sum of these two tests, ranging from 0 to 8.

The *z* scores were calculated and used to allow direct comparisons across different cognitive tests. Specifically, we standardized the follow-up score by subtracting the mean of the baseline score and then dividing it by the baseline standard deviation (SD). The global cognitive *z* score was estimated by averaging the *z* scores from the three tests and then standardizing it to baseline using the mean and SD of the global cognitive *z* score. Therefore, a unit of *z* score would mean the one SD above the mean baseline score.

### Statistical analysis

Baseline characteristics are presented as mean (SD) or median (interquartile range, IQR) for continuous variables and frequency (percentage) for categorical variables. Linear mixed models were used to investigate the difference in annual change of global cognitive function and specific cognitive domains per 10% increment in baseline FGCRS. We used the follow-up time (years since baseline) as a time scale. We fitted the models with the intercept and the time term as random effects accounting for inter-individual differences at baseline and changing rates in outcome variables during follow-up. For the fixed-effects part, we included baseline FGCRS, time, and baseline FGCRS × time interaction. The “baseline FGCRS × time” interaction term indicated a differential changing rate. We also categorized baseline FGCRS into tertiles and repeated the analysis using the low subgroup as the reference.

Change in FGCRS tertiles, assessed between baseline and 4 years after, yielded 9 possible combinations of FGCRS status: consistently low, low to intermediate, low to high, intermediate to low, consistently intermediate, intermediate to high, high to low, high to intermediate, and consistently high. Follow-up for cognitive decline still started from baseline. Then we investigated the difference in annual changes of cognitive decline among different FGCRS changing statuses using the consistently low group as the reference. The impact of continuous FGCRS change (per 10% increment in the difference) and its quintiles (using the middle quintile as the reference) on the cognitive decline was also estimated.

All models were adjusted for baseline covariates (age, sex, education, BMI, depression status, prevalent cardiovascular diseases) and baseline FGCRS score as appropriate. Given that the relationships of cardiovascular risk factors with cognition may vary with age (Armstrong et al., 2019), we tested the possible interaction by incorporating the “baseline FGCRS × time × baseline age” term into the model. Also, we repeated the main analyses after excluding those older than 75 years or with prevalent cardiovascular disease at baseline according to the FGCRS application recommendation (D’Agostino et al., 2008). In addition, to study the generalizability of our findings, external validation was performed using data from the English Longitudinal Study of Ageing (ELSA) (2008–2018), which is a nationally representative, biennial longitudinal survey of adults ≥ 50 years old residing in the United Kingdom (Steptoe et al., 2013), with similar statistical plan used. A more detailed description of the ELSA sample is provided in Supplementary material.

Data were handled and analyzed with SPSS Statistics version 25.0.0.1 (IBM Corp., Armonk, NY, United States) and R, CRAN version 4.1.2, with packages “lme4” and “lmerTest”. All analyses were performed at the significance level of 0.05 (2-tailed), unless specified.

## Results

### Participants’ characteristics

Among the 6,402 participants with complete baseline data and at least one reassessment of cognitive function, the mean (SD) age was 57.8 (8.4) years, 49.0% were women, the median (IQR) follow-up duration was 6.9 (4.0–7.0) years, and the median (IQR) number of cognitive assessments was 3 (Gorelick et al., 2017; World Health Organization, 2019; Livingston et al., 2020). Of those, 4,336 participants also had repeated FGCRS measurements. The distributions of baseline characteristics are shown in Table 1.

**TABLE 1:** Characteristics for the included participants at baseline and follow-up.

	Baseline FGCRS and cognitive decline	FGCRS change and cognitive decline
No. of participants	6,402	4,336
Follow-up time (years)	6.9 (4.0, 7.0)	6.9 (4.0, 7.0)
**Baseline status**		
Age at baseline (years)	57.8 (8.4)	57.8 (8.2)
Categorized baseline age		
45–54	2,435 (38.0%)	1,606 (37.0%)
55–64	2,588 (40.4%)	1,813 (41.8%)
65–74	1,151 (18.0%)	788 (18.2%)
75∼	228 (3.6%)	129 (3.0%)
Sex, female	3,140 (49.0%)	2,166 (50.0%)
Higher than high school	794 (12.4%)	489 (11.3%)
Body mass index (kg/m^2^)	23.7 (3.9)	23.8 (3.9)
Current smoker	2,074 (32.4%)	1,385 (31.9%)
Depression symptoms	1,601 (25.0%)	1,077 (24.8%)
Systolic blood pressure (mmHg)	129.0 (21.1)	128.4 (21.8)
Use of antihypertensive medication	1,238 (19.3%)	830 (19.1%)
Total cholesterol (mg/L)	193.9 (37.9)	193.5 (37.6)
HDL cholesterol (mg/L)	50.8 (15.2)	50.6 (15.2)
Prevalent diabetes	969 (15.1%)	652 (15.0%)
Prevalent cardiovascular disease	895 (14.0%)	602 (13.9%)
FGCRS (%)	17.0 (15.1)	16.8 (15.0)
Global cognition score	17 (13, 20)	17 (13, 20)
Orientation score	3 (3, 4)	3 (3, 4)
Memory score	7 (5, 10)	7 (5, 10)
Executive function score	7 (4, 8)	7 (4, 8)
**Follow-up status**		
Age (years)		61.8 (8.2)
Current smoker		1,220 (28.1%)
Systolic blood pressure (mmHg)		128.4 (20.3)
Use of antihypertensive medication		1,204 (27.8%)
Total cholesterol (mg/L)		185.8 (36.6)
HDL cholesterol (mg/L)		51.2 (12.0)
Prevalent diabetes		851 (19.6%)
FGCRS (%)		18.4 (15.2)

Values are mean (standard deviation) or median (interquartile range) for continuous variables and number (percentage) for categorical variables.

### Baseline Framingham General Cardiovascular Risk Score and subsequent cognitive decline

Table 2 and Supplementary Figure 1 demonstrate the annual change in cognition *z* scores among continuous and categorized FGCRS at baseline. A 10% increment in baseline FGCRS was associated with a faster decline in global cognition (−0.010 SD/year, 95% CI −0.013, −0.008), orientation (−0.006 SD/year, 95% CI −0.008, −0.003), memory (−0.014 SD/year, 95% CI −0.017, −0.010), and executive function (−0.005 SD/year, 95% CI −0.008, −0.002). When FGCRS was used as tertiles, the higher FGCRS was still associated with faster cognitive decline than the low tertile. For example, compared to those within the low FGCRS tertile, those within the high FGCRS tertile had significantly worse performance in global cognition (−0.035 SD/year, 95% CI −0.045, −0.026), orientation (−0.014 SD/year, 95% CI −0.025, −0.004), memory (−0.048 SD/year, 95% CI −0.060, −0.036), and executive function (−0.021 SD/year, 95% CI −0.031, −0.011).

**TABLE 2:** The associations between baseline Framingham General Cardiovascular Risk Score (FGCRS) and annual changes in cognition *z* scores (SD/year), using linear mixed models.

Framingham General Cardiovascular Risk	No.	Global cognitive *z* score	Orientation *z* score	Memory *z* score	Executive function *z* score
		β (95% CI)	β (95% CI)	β (95% CI)	β (95% CI)
Continuous FGCRS	6,402	−0.01 (−0.013, −0.008)	−0.006 (−0.008, −0.003)	−0.014 (−0.017, −0.01)	−0.005 (−0.008, −0.002)
**FGCRS categories**					
Low (<8.0%)	2,134	Reference	Reference	Reference	Reference
Intermediate (8.0–18.3%)	2,134	−0.015 (−0.025, −0.006)	0.006 (−0.004, 0.017)	−0.028 (−0.039, −0.017)	−0.012 (−0.022, −0.002)
High (>18.3%)	2,134	−0.035 (−0.045, −0.026)	−0.014 (−0.025, −0.004)	−0.048 (−0.06, −0.036)	−0.021 (−0.031, −0.011)

β represents cognition *z* scores (dependent variables) as a function of FGCRS (as a continuous or categorized variable). Each SD change of cognition *z* score responded to the change per 0.1 increment in FGCRS when it was a continuous variable. When the FGCRS was a categorical variable (tertiles), β represents each unit (SD) in cognition *z* scores varied by per tertile (intermediate/high) in FGCRS compared with the low.

Analyses were adjusted for baseline age, sex, education level, body mass index, depression status, and prevalent cardiovascular diseases (heart disease, stroke).

### Change in Framingham General Cardiovascular Risk Score and concomitant cognitive decline

**FIGURE 2:**
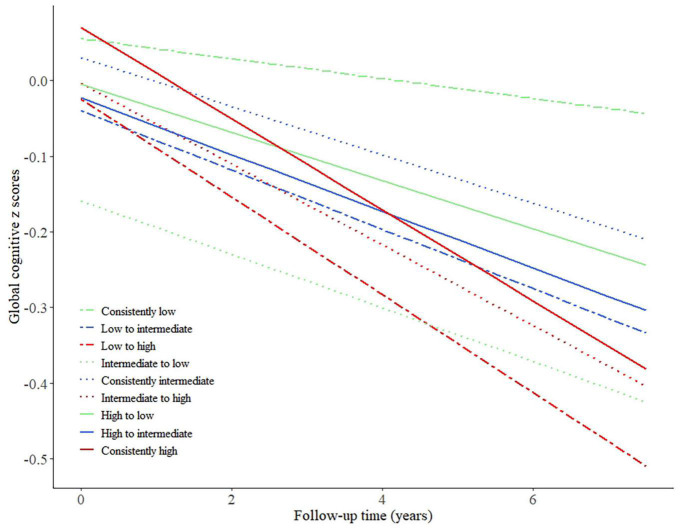
Predicted trajectories of global cognitive *z* scores by patterns of change in Framingham General Cardiovascular Risk Score between baseline and 4 years after, adjusted for baseline age, sex, education level, body mass index, depression status, prevalent cardiovascular diseases (heart disease, stroke).

The associations between change in FGCRS and cognitive decline are reported in Table 3, Figure 2, and Supplementary Figure 2. Among the nine combinations of FGCRS changing status, two included very few subjects (36 for the low to high group; 13 for the high to low group). In multivariable linear mixed model analysis, the rate of annual global cognitive decline was not statistically different from that of the consistently low group (reference) in the intermediate to low group (−0.022 SD/year, 95% CI −0.050, 0.005) and the high to low group (−0.019 SD/year, 95% CI −0.103, 0.066). Instead, the annual global cognitive decline was faster in the low to intermediate group (−0.026 SD/year, 95% CI −0.045, −0.007), the low to high group (−0.052 SD/year, 95% CI −0.102, −0.001), the consistently intermediate group (−0.019 SD/year, 95% CI −0.033, −0.005), the intermediate to high group (−0.040 SD/year, 95% CI −0.058, −0.022), the high to intermediate group (−0.024 SD/year, 95% CI −0.047, −0.002), and the consistently high group (−0.047 SD/year, 95% CI −0.060, −0.034). In addition, there was no significant faster global cognitive decline in difference in continuous FGCRS (10% increment of difference: −0.001, 95% CI −0.006, 0.004). When a change in continuous FGCRS was used as a quantile, the lower and higher FGCRS quintiles were significantly associated with faster cognitive decline than the middle quintile. For example, compared to those with middle FGCRS, those within the lowest and highest FGCRS quintiles had significantly worse performance in global cognition (−0.028 SD/year, 95% CI −0.043, −0.013 for the lowest quintile; −0.032 SD/year, 95% CI −0.047, −0.017 for the highest quintile). The results were similar for specific cognitive domains.

**TABLE 3:** The associations between change in Framingham General Cardiovascular Risk Score (FGCRS) and annual changes in cognition *z* scores (SD/year), using linear mixed models.

Framingham General Cardiovascular Risk	No.	Global cognitive *z* score	Orientation *z* score	Memory *z* score	Executive function *z* score
		β (95% CI)	β (95% CI)	β (95% CI)	β (95% CI)
**Change in FGCRS status**					
Consistently low	1,092	Reference	Reference	Reference	Reference
Low to intermediate	355	−0.026 (−0.045, −0.007)	−0.004 (−0.025, 0.016)	−0.024 (−0.046, −0.002)	−0.027 (−0.047, −0.007)
Low to high	36	−0.052 (−0.102, −0.001)	−0.066 (−0.123, −0.01)	−0.049 (−0.111, 0.013)	0.011 (−0.042, 0.064)
Intermediate to low	148	−0.022 (−0.050, 0.005)	0.008 (−0.021, 0.038)	−0.028 (−0.060, 0.005)	−0.028 (−0.057, 0.001)
Consistently intermediate	870	−0.019 (−0.033, −0.005)	0.008 (−0.007, 0.023)	−0.035 (−0.052, −0.019)	−0.014 (−0.028, 0.001)
Intermediate to high	418	−0.040 (−0.058, −0.022)	−0.011 (−0.030, 0.009)	−0.044 (−0.065, −0.022)	−0.029 (−0.048, −0.011)
High to low	13	−0.019 (−0.103, 0.066)	−0.02 (−0.115, 0.075)	−0.068 (−0.172, 0.036)	0.026 (−0.064, 0.116)
High to intermediate	227	−0.024 (−0.047, −0.002)	0.002 (−0.023, 0.027)	−0.029 (−0.057, −0.002)	−0.034 (−0.058, −0.010)
Consistently high	1,177	−0.047 (−0.060, −0.034)	−0.02 (−0.034, −0.006)	−0.057 (−0.072, −0.041)	−0.026 (−0.039, −0.012)
**Change in continuous FGCRS**					
Per 10% increment in score difference	4,336	−0.001 (−0.006, 0.004)	−0.002 (−0.008, 0.004)	−0.002 (−0.008, 0.004)	−0.001 (−0.006, 0.004)
Quintile 1 (<−2.8%)	868	−0.028 (−0.043, −0.013)	−0.004 (−0.021, 0.012)	−0.040 (−0.057, −0.022)	−0.016 (−0.031, 0.000)
Quintile 2 (−2.8 to 0.3%)	867	−0.009 (−0.023, 0.006)	0.000 (−0.016, 0.017)	−0.010 (−0.028, 0.008)	−0.004 (−0.020, 0.011)
Quintile 3 (0.3–2.3%)	867	Reference	Reference	Reference	Reference
Quintile 4 (2.3–6.5%)	867	−0.014 (−0.029, 0.000)	−0.002 (−0.018, 0.014)	−0.024 (−0.041, −0.006)	−0.005 (−0.021, 0.010)
Quintile 5 (>6.5%)	867	−0.032 (−0.047, −0.017)	−0.013 (−0.029, 0.003)	−0.042 (−0.060, −0.025)	−0.015 (−0.031, 0.001)

Analyses were adjusted for baseline age, sex, education level, body mass index, depression status, and prevalent cardiovascular diseases (heart disease, stroke). Analyses for change in continuous FGCRS were additionally adjusted for baseline FGCRS score.

### Sensitivity/additional analyses

No significant interaction between baseline FGCRS and age was observed in the changes in cognitive decline (*p* for “baseline FGCRS × time × baseline age” term: 0.692 for global cognition, 0.507 for orientation, 0.127 for memory, and 0.362 for executive function). Similar associations between baseline FGCRS and its change with cognitive decline were observed after excluding participants with prevalent cardiovascular disease or aged above 75 years at baseline (Supplementary Tables 1, 2). When the external validation was performed using data from the ELSA, the results remained consistent with our main findings (Supplementary Tables 3, 4).

### Non-response analyses

As shown in Figure 1, among the 7,166 participants who had complete FGCRS and cognition measurements at baseline, 764 (10.7%) were excluded from the baseline FGCRS analysis because of missing information or loss of follow-up. Compared to the included participants, those excluded participants were older, more often women, less educated, and had worse cardiovascular risk profiles at baseline. 2,066 (32.3%) out of 6,402 participants were further excluded due to unavailable FGCRS reassessment. Those included and excluded from the FGCRS change analysis shared similar baseline characteristics (Supplementary Table 5).

## Discussion

In this longitudinal analysis of 6,402 community-dwelling middle-aged and older adults from the CHARLS, we found that increased cardiovascular risk burden measured by baseline FGCRS was associated with a faster decline in global cognition and specific cognitive domains during follow-up. However, there was no consistent relationship between the direction of changes in FGCRS and later cognitive decline.

The association of composite cardiovascular risk score with cognitive decline has been investigated from a life-course perspective (Kaffashian et al., 2011; Yaffe et al., 2014, 2021; George et al., 2021), but with inconsistent results, especially for the exposure during older age. The Framingham Offspring Study showed that cardiovascular risk burden during midlife, but not late-life, was associated with faster cognitive decline (Armstrong et al., 2019). Prior neuroimaging studies also found that only midlife, but not late-life, vascular risk factors were associated with elevated brain amyloid and lower gray matter perfusion at older ages (Gottesman et al., 2017; Suri et al., 2019). However, one recent study based on the Rush Memory and Aging Project reported that among participants with an average age of 80 years, a higher cardiovascular risk burden can still predict a decline in episodic memory, working memory, and perceptual speed and is associated with neurodegeneration and vascular lesions in the brain (Song et al., 2020). Here among the middle-aged and older Chinese participants, we found that increased cardiovascular risk burden was associated with faster cognitive decline. This association was not modified by baseline age. The differences between the findings may be explained by the heterogeneity in cohort characteristics (e.g., ethnicity, education level), and methodological discrepancies, such as duration of follow-up and the tools for cognitive assessment. Although the 95% CIs of global cognition decline would meet the clinical threshold of cognition change, a decline of ≥0.5 SD (Norman et al., 2003), in approximately 10 years since baseline, even minuscule cognitive function decline can result in a substantially increased risk of dementia over a long-term (Bozoki et al., 2001). Our findings support careful monitoring for cognitive impairment among patients with a higher cardiovascular risk burden.

The present study is the first prospective investigation of the cognitive trajectories among different patterns of change in composite cardiovascular risk scores. Previous studies mainly documented changes in specific cardiovascular risk factors such as BMI, blood pressure with cognitive decline, and dementia onset (Walker et al., 2019; Wu et al., 2021). The Atherosclerosis Risk in Communities study found that sustained hypertension in midlife to late-life and a pattern of midlife hypertension and late-life hypotension, compared with midlife and late-life normal blood pressure, were associated with increased risk for subsequent dementia. In contrast, no significant association was found between 24-year blood pressure patterns and cognitive change in late life (Walker et al., 2019). Here we calculated the changing pattern of FGCRS within 4 years among middle-aged and older Chinese participants and found no consistent relationship between the direction of changes in FGCRS and cognitive decline. These inconsistent relationship patterns indicate that the concept of FGCRS may not be useful for assessing the change in cardiovascular risk burden over time. A composite score cannot simply capture the complex relationship between cardiovascular risk factors and cognitive decline. The disease prognosis may be determined primarily by early exposure to a high cardiovascular risk burden, which may explain that participants who changed from high FGCRS to a lower category still had a relatively faster cognitive decline.

The current study has several strengths. The CHARLS is well designed to provide a nationally representative estimate for middle-aged and older people in China. The data on biomarkers were collected following standardized protocols with high quality. Additionally, with repeated data of FGCRS during follow-up, we explored any possible effect caused by different changing patterns of FGCRS. Furthermore, our findings can be generalized to Western populations by externally validating our results using the ELSA. Taken together, our study filled in a specific knowledge gap about the association between changes in FGCRS and cognitive decline. However, our study also has some limitations that should be acknowledged. First, limited by the available waves in the CHARLS, the follow-up interval was relatively short, and we could not investigate the association of changes in FGCRS with subsequent cognitive decline in our main analysis. However, since results from the external validation using the ELSA data with cognition measured from wave 6 onward remained consistent, it is unlikely that our main findings would be biased by reverse causation. Second, genetic data were unavailable, so we could not adjust for the APOE genotype; however, previous studies indicated no interaction between FGCRS and APOE status on cognitive decline (Armstrong et al., 2019; Song et al., 2020). Meanwhile, only those with complete FGCRS information and at least one repeated measurement were eligible for the current study, leading possibly to selection bias. The non-response analysis showed that the included participants were relatively healthier than those excluded, which may limit the internal validity. Moreover, our analysis of responders’ data may have underestimated complications by excluding non-responders’ potentially faster cognitive decline.

In summary, we found that a higher cardiovascular risk burden assessed by baseline FGCRS was associated with faster cognitive decline among the middle-aged and older Chinese population. However, there was no consistent relationship between the direction of change in FGCRS and cognitive decline. Further studies are needed to determine whether decreasing FGCRS would benefit cognitive decline.

## Data availability statement

The raw data supporting the conclusions of this article will be made available by the authors, without undue reservation.

## Ethics statement

The studies involving human participants were reviewed and approved by the Peking University Institutional Review Board (for the CHARLS) and the London Multicenter Research Ethics Committee (for the ELSA). The patients/participants provided their written informed consent to participate in this study.

## Author contributions

XJ, HG, KW, and FA were responsible for the study concept and design. KW composed the statistical dataset, performed the statistical analyses, guarantor of this work and had full access to all data in the study, and took responsibility for the integrity of the data and the accuracy of the data analysis. XJ and HG wrote the manuscript. All authors contributed to the interpretation of the data and critical revision of the manuscript.
