# Supplementary material for: Association of baseline level of cardiovascular risk burden and its temporal changes with cognitive decline
Source: Front Aging Neurosci. 2022 Sep 1;14:895188. doi: 10.3389/fnagi.2022.895188 (PMC9474998; doi:10.3389/fnagi.2022.895188)

**Association of baseline level of cardiovascular risk burden and its temporal changes with cognitive decline**

**Supplement materials**

**External validation (the ELSA)**

***Study population***

For the external validation, we used data from wave 4 to wave 9 (2008 to 2018) of the English Longitudinal Study of Ageing (ELSA), a community-based longitudinal cohort conducted in the United Kingdom. The ELSA was approved by the London Multicenter Research Ethics Committee, and all participants provided written informed consent. A total of 6036 participants had physical and clinical data at baseline; 863 were excluded: younger than 45 years (n=12), self-reported doctor-diagnosed mental disease (n=28), unavailable information to assess FGCRS (n=43) or cognitive function (n=13) at baseline, loss to follow-up (n=480), or missing covariates at baseline (n=287). Hence, a total of 5173 participants from the ELSA with complete measurements of FGCRS and cognitive function at baseline and at least one reassessment of cognitive function during 4 years of follow-up were included in our analysis. Of these, 3494 participants had data about FGCRS at wave 6 (4 years after baseline).

***Cognitive assessments***

In the ELSA, the cognitive assessment was performed in all waves, including three domains: orientation, memory, and executive function. Orientation was measured by asking four questions regarding the date (year, month, day of month, and day of week). One point was given for each correct answer, with the sum score ranging from 0 to 4. The memory assessment comprised immediate and delayed recall for ten unrelated words. The sum of words successfully recalled in these two tests, was used and ranged from 0 to 20. The executive function in the ELSA was assessed differently from the CHARLS, participants were asked to name as many animals as they could in one minute, and then the number of animal names was counted as the executive score. Then, the *z* scores were also calculated and used.

***Statistical analysis***

For the external validation, we only investigated the association between change in FGCRS and cognitive decline using the ELSA. Similarly, the temporal patterns of FGCRS tertiles, assessed between baseline and 4 years after (wave 4 and wave 6 for the ELSA), yielded 9 combinations: consistently low, low to intermediate, low to high, intermediate to low, consistently intermediate, intermediate to high, high to low, high to intermediate, and consistently high. Follow-up for cognitive decline started from baseline (wave 4 for the ELSA). Then we investigated the difference in annual changes of cognitive decline among different FGCRS changing statuses using the consistently low group as the reference. The impact of continuous FGCRS change (per 10% increment in the difference) and its quintiles (using the middle quintile as the reference) on the cognitive decline was also estimated. In addition, we investigated the association between change in FGCRS (wave 4 to wave 6) and subsequent cognitive decline (started from wave 6) in the ELSA (n=3185). All models were adjusted for baseline covariates (age, sex, education, BMI, depression status, prevalent cardiovascular diseases), and baseline FGCRS score as appropriate.

**ESM Table 1**. The associations between baseline Framingham General Cardiovascular Risk Score (FGCRS) and annual changes in cognition *z* scores (SD/year), excluding those older than 75 years or with prevalent cardiovascular disease at baseline

| **Framingham General Cardiovascular Risk** | | **Global cognitive *Z* score** | **Orientation *Z* score** | **Memory *Z* score** | **Executive function *Z* score** |
| --- | --- | --- | --- | --- | --- |
|  |  | **β (95%CI)** | **β (95%CI)** | **β (95%CI)** | **β (95%CI)** |
| Continuous FGCRS | | -0.01 (-0.014, -0.007) | -0.005 (-0.008, -0.001) | -0.013 (-0.017, -0.01) | -0.005 (-0.008, -0.002) |
| FGCRS categories | |  |  |  |  |
|  | Low | Reference | Reference | Reference | Reference |
|  | Intermediate | -0.018 (-0.028, -0.008) | 0.007 (-0.004, 0.018) | -0.032 (-0.044, -0.02) | -0.012 (-0.023, -0.001) |
|  | High | -0.034 (-0.045, -0.024) | -0.013 (-0.024, -0.001) | -0.047 (-0.06, -0.034) | -0.022 (-0.033, -0.01) |

β represents cognition *z* scores (dependent variables) as a function of FGCRS (as a continuous or categorized variable). Each point change of cognition *z* score responded to the change of per 0.10 increment in FGCRS when it was a continuous variable. When the FGCRS was a categorical variable (tertiles), β represents each score in cognition *z* scores varied by per tertile (intermediate/high) in FGCRS compared with the low.

Analyses were adjusted for baseline age, sex, education level, body mass index, and depression status.

**ESM Table 2**. The associations between change in Framingham General Cardiovascular Risk Score (FGCRS) and annual changes in cognition *z* scores (SD/year), excluding those older than 75 years or with prevalent cardiovascular disease at baseline

| **Framingham General Cardiovascular Risk** | | **Global cognitive *Z* score** | **Orientation *Z* score** | **Memory *Z* score** | **Executive function *Z* score** |
| --- | --- | --- | --- | --- | --- |
|  |  | **β (95%CI)** | **β (95%CI)** | **β (95%CI)** | **β (95%CI)** |
| **Change in FGCRS status** | |  |  |  |  |
|  | Consistently low | Reference | Reference | Reference | Reference |
|  | Low to intermediate | -0.023 (-0.043, -0.003) | 0 (-0.021, 0.021) | -0.024 (-0.047, 0) | -0.028 (-0.049, -0.007) |
|  | Low to high | -0.041 (-0.095, 0.013) | -0.067 (-0.128, -0.005) | -0.029 (-0.097, 0.038) | 0.02 (-0.037, 0.077) |
|  | Intermediate to low | -0.025 (-0.055, 0.005) | 0 (-0.032, 0.031) | -0.024 (-0.059, 0.011) | -0.028 (-0.059, 0.003) |
|  | Consistently intermediate | -0.018 (-0.033, -0.003) | 0.011 (-0.005, 0.027) | -0.039 (-0.056, -0.021) | -0.012 (-0.028, 0.003) |
|  | Intermediate to high | -0.045 (-0.064, -0.026) | -0.009 (-0.03, 0.012) | -0.052 (-0.075, -0.029) | -0.029 (-0.049, -0.009) |
|  | High to low | -0.015 (-0.106, 0.076) | -0.018 (-0.118, 0.082) | -0.074 (-0.185, 0.037) | 0.026 (-0.071, 0.124) |
|  | High to intermediate | -0.011 (-0.036, 0.013) | 0.015 (-0.012, 0.042) | -0.029 (-0.059, 0.001) | -0.028 (-0.055, -0.002) |
|  | Consistently high | -0.046 (-0.06, -0.031) | -0.019 (-0.034, -0.003) | -0.055 (-0.072, -0.038) | -0.024 (-0.039, -0.009) |
| **Change in continuous FGCRS** | |  |  |  |  |
|  | Per 10% increament in score difference | -0.004 (-0.009, 0.002) | -0.003 (-0.01, 0.003) | -0.005 (-0.012, 0.002) | 0 (-0.006, 0.006) |
|  | Quintile 1 | -0.026 (-0.042, -0.009) | -0.001 (-0.018, 0.017) | -0.035 (-0.054, -0.015) | -0.016 (-0.033, 0.001) |
|  | Quintile 2 | -0.008 (-0.023, 0.008) | 0.002 (-0.015, 0.019) | -0.008 (-0.027, 0.01) | -0.005 (-0.021, 0.012) |
|  | Quintile 3 | Reference | Reference | Reference | Reference |
|  | Quintile 4 | -0.014 (-0.029, 0.002) | 0.003 (-0.014, 0.02) | -0.023 (-0.042, -0.004) | -0.006 (-0.023, 0.011) |
|  | Quintile 5 | -0.036 (-0.052, -0.019) | -0.009 (-0.027, 0.009) | -0.049 (-0.068, -0.029) | -0.016 (-0.033, 0.001) |

Analyses were adjusted for baseline age, sex, education level, body mass index, and depression status. Analyses for change in continuous FGCRS were additionally adjusted for baseline FGCRS score.

**ESM Table 3**. The associations between change in Framingham General Cardiovascular Risk Score (FGCRS) and concomitant annual changes in cognition *z* scores (SD/year), external validation using the ELSA

| **Framingham General Cardiovascular Risk** | | **No.** | **Global cognitive *Z* score** | **Orientation *Z* score** | **Memory *Z* score** | **Executive function *Z* score** |
| --- | --- | --- | --- | --- | --- | --- |
|  |  |  | **β (95%CI)** | **β (95%CI)** | **β (95%CI)** | **β (95%CI)** |
| **Change in FGCRS status** | |  |  |  |  |  |
|  | Consistently low | 855 | Reference | Reference | Reference | Reference |
|  | Low to intermediate | 343 | -0.004 (-0.019, 0.011) | 0 (-0.021, 0.02) | 0 (-0.012, 0.012) | -0.011 (-0.023, 0.001) |
|  | Low to high | 22 | -0.042 (-0.092, 0.007) | -0.045 (-0.113, 0.024) | -0.02 (-0.06, 0.02) | -0.02 (-0.061, 0.021) |
|  | Intermediate to low | 138 | -0.035 (-0.056, -0.013) | -0.043 (-0.072, -0.014) | -0.016 (-0.033, 0.002) | -0.022 (-0.04, -0.004) |
|  | Consistently intermediate | 691 | -0.017 (-0.029, -0.005) | -0.02 (-0.037, -0.004) | -0.01 (-0.02, -0.001) | -0.007 (-0.017, 0.003) |
|  | Intermediate to high | 340 | -0.039 (-0.054, -0.024) | -0.038 (-0.059, -0.017) | -0.02 (-0.032, -0.008) | -0.022 (-0.034, -0.009) |
|  | High to low | 14 | -0.008 (-0.074, 0.057) | -0.056 (-0.146, 0.035) | -0.004 (-0.058, 0.049) | 0.036 (-0.019, 0.091) |
|  | High to intermediate | 187 | -0.051 (-0.071, -0.032) | -0.066 (-0.093, -0.039) | -0.023 (-0.039, -0.007) | -0.032 (-0.048, -0.016) |
|  | Consistently high | 904 | -0.041 (-0.052, -0.03) | -0.03 (-0.045, -0.014) | -0.03 (-0.039, -0.021) | -0.031 (-0.04, -0.021) |
| **Change in continuous FGCRS** | |  |  |  |  |  |
|  | Per 10% increment in score difference | 3494 | 0.002 (-0.004, 0.007) | 0.005 (-0.003, 0.012) | 0.002 (-0.003, 0.006) | 0.001 (-0.004, 0.005) |
|  | Quintile 1 (< -2.5%) | 699 | -0.032 (-0.045, -0.019) | -0.038 (-0.055, -0.02) | -0.019 (-0.03, -0.009) | -0.02 (-0.03, -0.009) |
|  | Quintile 2 (-2.5% ~ 0.4%) | 699 | -0.007 (-0.02, 0.005) | -0.017 (-0.035, 0) | 0 (-0.01, 0.01) | 0.002 (-0.008, 0.012) |
|  | Quintile 3 (0.4% ~ 2.5%) | 699 | Reference | Reference | Reference | Reference |
|  | Quintile 4 (2.5% ~ 5.9%) | 699 | -0.003 (-0.015, 0.01) | -0.005 (-0.022, 0.012) | 0.006 (-0.004, 0.017) | -0.007 (-0.017, 0.004) |
|  | Quintile 5 (> 5.9%) | 698 | -0.026 (-0.038, -0.013) | -0.033 (-0.05, -0.015) | -0.013 (-0.023, -0.003) | -0.01 (-0.021, 0) |

Analyses were adjusted for baseline age, sex, education level, body mass index, depression status, and prevalent cardiovascular diseases (heart disease, stroke). Analyses for change in continuous FGCRS were additionally adjusted for baseline FGCRS score.

**ESM Table 4**. The association between change in Framingham General Cardiovascular Risk Score (FGCRS) and subsequent annual changes in cognition *z* scores (SD/year), external validation using the ELSA

| **Framingham General Cardiovascular Risk** | | **No.** | **Global cognitive *z* score** | **Orientation *z* score** | **Memory *z* score** | **Executive function *z* score** |
| --- | --- | --- | --- | --- | --- | --- |
|  |  |  | **β (95%CI)** | **β (95%CI)** | **β (95%CI)** | **β (95%CI)** |
| **Change in FGCRS status** | |  |  |  |  |  |
|  | Consistently low | 750 | Reference | Reference | Reference | Reference |
|  | Low to intermediate | 297 | -0.016 (-0.041, 0.010) | -0.008 (-0.044, 0.029) | -0.005 (-0.026, 0.015) | -0.016 (-0.038, 0.006) |
|  | Low to high | 25 | -0.025 (-0.099, 0.049) | -0.036 (-0.143, 0.071) | 0.010 (-0.051, 0.071) | -0.024 (-0.088, 0.040) |
|  | Intermediate to low | 128 | -0.036 (-0.072, 0.000) | -0.043 (-0.095, 0.009) | -0.005 (-0.035, 0.024) | -0.039 (-0.07, -0.008) |
|  | Consistently intermediate | 634 | -0.009 (-0.029, 0.011) | -0.009 (-0.038, 0.020) | -0.008 (-0.025, 0.008) | 0.001 (-0.017, 0.018) |
|  | Intermediate to high | 307 | -0.061 (-0.087, -0.035) | -0.061 (-0.098, -0.024) | -0.028 (-0.049, -0.007) | -0.028 (-0.05, -0.006) |
|  | High to low | 12 | -0.041 (-0.154, 0.073) | -0.054 (-0.217, 0.110) | 0.002 (-0.091, 0.096) | -0.045 (-0.143, 0.053) |
|  | High to intermediate | 155 | -0.055 (-0.089, -0.021) | -0.059 (-0.108, -0.010) | -0.018 (-0.046, 0.010) | -0.045 (-0.075, -0.016) |
|  | Consistently high | 877 | -0.057 (-0.076, -0.038) | -0.052 (-0.080, -0.025) | -0.03 (-0.045, -0.014) | -0.04 (-0.056, -0.024) |
| **Change in continuous FGCRS** | |  |  |  |  |  |
|  | Per 10% increment in score difference | 3185 | -0.005 (-0.014, 0.005) | -0.005 (-0.018, 0.009) | 0.000 (-0.008, 0.008) | -0.002 (-0.01, 0.006) |
|  | Quintile 1 (< -2.4%) | 637 | -0.035 (-0.056, -0.013) | -0.04 (-0.071, -0.009) | -0.016 (-0.033, 0.002) | -0.026 (-0.044, -0.007) |
|  | Quintile 2 (-2.4% ~ 0.4%) | 637 | -0.003 (-0.024, 0.018) | -0.020 (-0.051, 0.010) | 0.006 (-0.012, 0.023) | 0.008 (-0.01, 0.026) |
|  | Quintile 3 (0.4% ~ 2.5%) | 637 | Reference | Reference | Reference | Reference |
|  | Quintile 4 (2.5% ~ 5.8%) | 637 | -0.012 (-0.033, 0.010) | -0.019 (-0.049, 0.012) | 0.003 (-0.014, 0.021) | -0.007 (-0.025, 0.011) |
|  | Quintile 5 (> 5.8%) | 637 | -0.037 (-0.059, -0.016) | -0.058 (-0.089, -0.028) | -0.011 (-0.029, 0.006) | -0.013 (-0.031, 0.006) |

Analyses were adjusted for baseline age, sex, education level, body mass index, depression status, and prevalent cardiovascular diseases (heart disease, stroke). Analyses for change in continuous FGCRS were additionally adjusted for baseline FGCRS score.

**ESM Table 5**. Comparison of baseline characteristics between participants included and excluded

|  | | **Baseline FGCRS & Cognitive decline** | | **FGCRS Change & Cognitive decline** | |
| --- | --- | --- | --- | --- | --- |
|  |  | **Excluded** | **Included** | **Excluded** | **Included** |
| **No. of participants** | | 764 | 6402 | 2066 | 4336 |
| **Baseline status** | |  |  |  |  |
|  | Age at baseline, years | 63.1 (10.9) | 57.8 (8.4) | 57.9 (8.8) | 57.8 (8.2) |
|  | Sex, female | 422 (55.2%) | 3140 (49.0%) | 974 (47.1%) | 2166 (50.0%) |
|  | Higher than high school | 51 (6.7%) | 794 (12.4%) | 305 (14.8%) | 489 (11.3%) |
|  | Body mass index, kg/m^2^ | 23.0 (4.0) | 23.7 (3.9) | 23.6 (3.9) | 23.8 (3.9) |
|  | Current smoker | 237 (31.0%) | 2074 (32.4%) | 689 (33.3%) | 1385 (31.9%) |
|  | Depression symptoms | 274 (35.9%) | 1601 (25.0%) | 524 (25.4%) | 1077 (24.8%) |
|  | Systolic blood pressure, mmHg | 132.5 (22.2) | 129.0 (21.1) | 130.1 (21.6) | 128.4 (21.8) |
|  | Use of antihypertensive medication | 176 (23.0%) | 1238 (19.3%) | 408 (19.7%) | 830 (19.1%) |
|  | Total cholesterol, mg/L | 191.5 (38.9) | 193.9 (37.9) | 194.7 (38.3) | 193.5 (37.6) |
|  | HDL cholesterol, mg/L | 51.0 (14.5) | 50.8 (15.2) | 51.1 (15.2) | 50.6 (15.2) |
|  | Prevalent diabetes | 135 (17.7%) | 969 (15.1%) | 317 (15.3%) | 652 (15.0%) |
|  | Prevalent cardiovascular disease | 119 (16.0%) | 895 (14.0%) | 293 (14.2%) | 602 (13.9%) |
|  | FGCRS, % | 21.0 (17.9) | 17.0 (15.1) | 17.5 (15.3) | 16.8 (15.0) |
| **4-years after** | |  |  |  |  |
|  | Age at follow-up, years |  |  | 61.7 (8.8) | 61.8 (8.2) |
|  | Current smoker |  |  | 500 (30.5%) | 1220 (28.1%) |
|  | Systolic blood pressure, mmHg |  |  | 127.3 (20.1) | 128.4 (20.3) |
|  | Use of antihypertensive medication |  |  | 372 (26.3%) | 1204 (27.8%) |
|  | Total cholesterol, mg/L |  |  | 186.4 (43.4) | 185.8 (36.6) |
|  | HDL cholesterol, mg/L |  |  | 51.4 (11.9) | 51.2 (12.0) |
|  | Prevalent diabetes |  |  | 207 (13.0%) | 851 (19.6%) |
|  | FGCRS, % |  |  | NA | 18.4 (15.2) |

Values are mean (standard deviation) for continuous variables and number (percentage) for categorical variables.

**ESM Figure 1**. Predicted trajectories of cognitive *z* scores by baseline FGCRS tertiles, adjusted for baseline age, sex, education level, body mass index, depression, and prevalent cardiovascular diseases


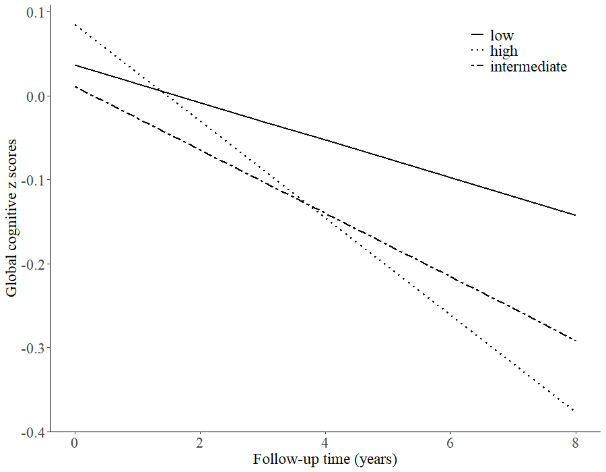

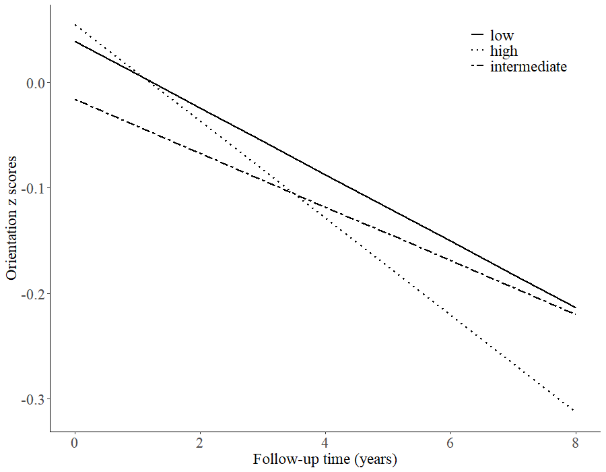


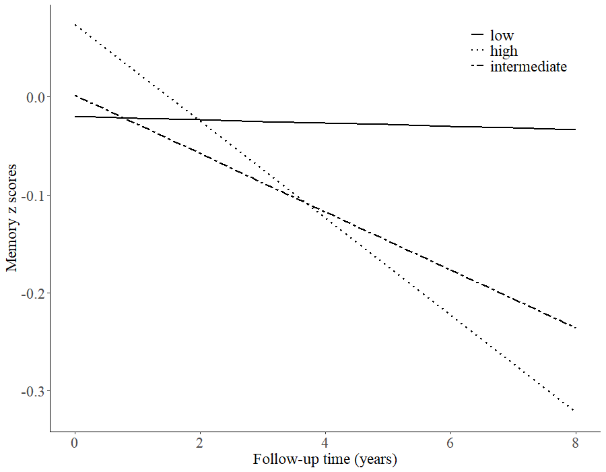

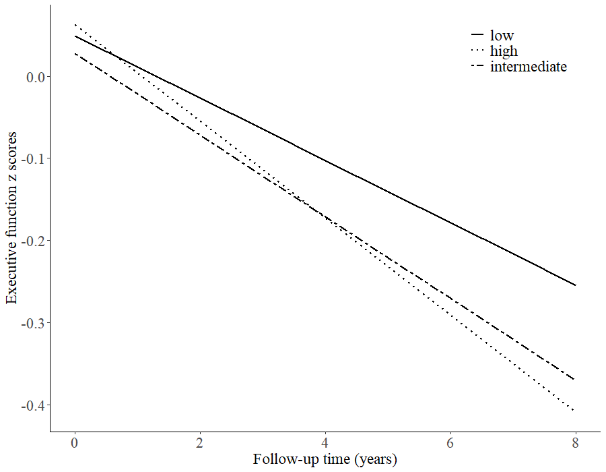


**ESM Figure 2**. Predicted trajectories of specific cognitive domains by patterns of change in FGCRS between baseline and 4 years after, adjusted for baseline age, sex, education level, body mass index, depression, and prevalent cardiovascular diseases


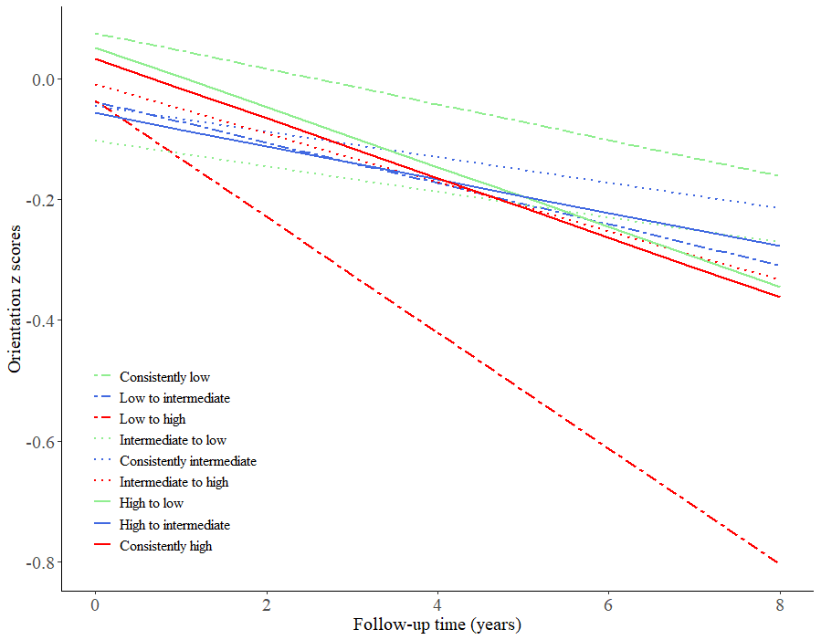


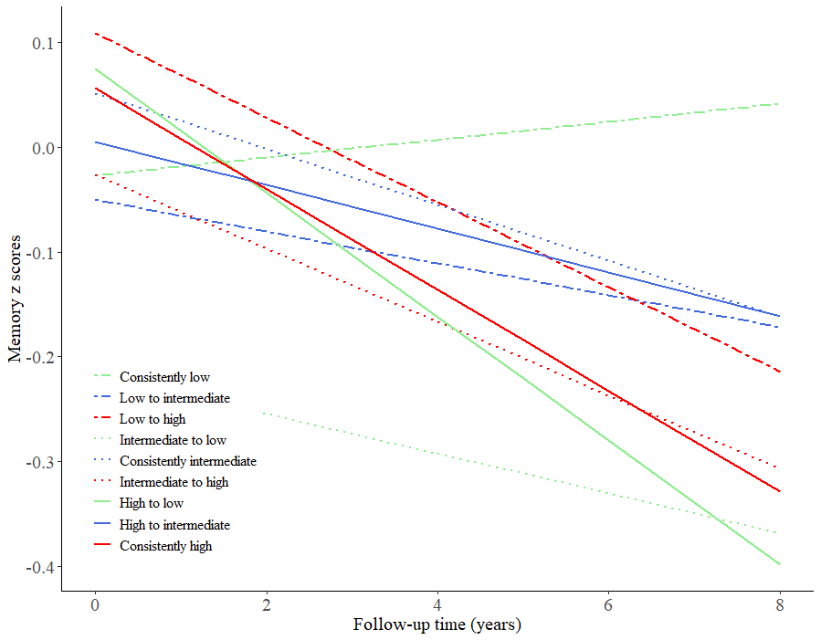


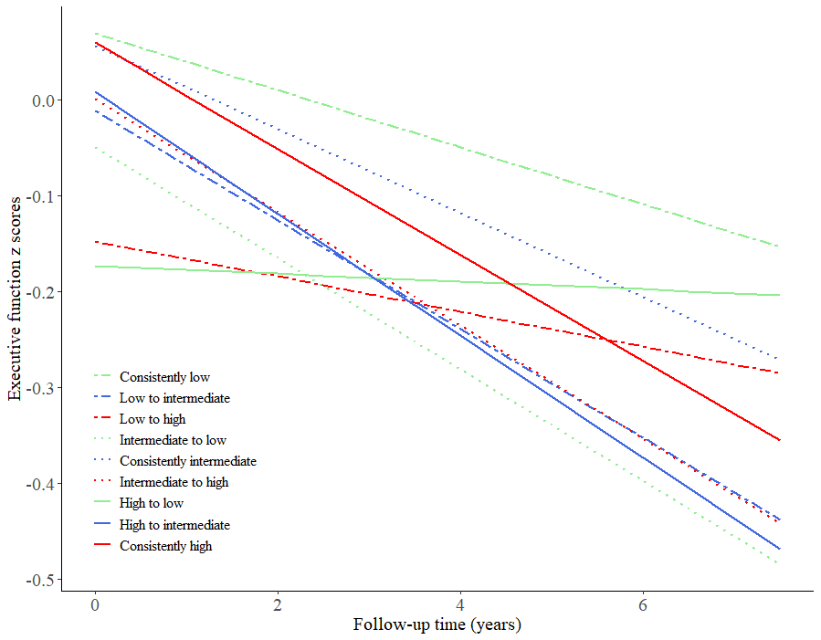

Supplement: Supplementary file 1 [file Data_Sheet_1.docx]
